# Keeping in Touch with One's Self: Multisensory Mechanisms of Self-Consciousness

**DOI:** 10.1371/journal.pone.0006488

**Published:** 2009-08-05

**Authors:** Jane E. Aspell, Bigna Lenggenhager, Olaf Blanke

**Affiliations:** 1 Laboratory of Cognitive Neuroscience, Ecole Polytechnique Fédérale de Lausanne (EPFL), Lausanne, Switzerland; 2 Department of Neurology, University Hospital, Geneva, Switzerland; Macquarie University, Australia

## Abstract

**Background:**

The spatial unity between self and body can be disrupted by employing conflicting visual-somatosensory bodily input, thereby bringing neurological observations on bodily self-consciousness under scientific scrutiny. Here we designed a novel paradigm linking the study of bodily self-consciousness to the spatial representation of visuo-tactile stimuli by measuring crossmodal congruency effects (CCEs) for the full body.

**Methodology/Principal Findings:**

We measured full body CCEs by attaching four vibrator-light pairs to the trunks (backs) of subjects who viewed their bodies from behind via a camera and a head mounted display (HMD). Subjects made speeded elevation (up/down) judgments of the tactile stimuli while ignoring light stimuli. To modulate self-identification for the seen body subjects were stroked on their backs with a stick and the felt stroking was either synchronous or asynchronous with the stroking that could be seen via the HMD.

We found that (1) tactile stimuli were mislocalized towards the seen body (2) CCEs were modulated systematically during visual-somatosensory conflict when subjects viewed their body but not when they viewed a body-sized object, i.e. CCEs were larger during synchronous than during asynchronous stroking of the body and (3) these changes in the mapping of tactile stimuli were induced in the same experimental condition in which predictable changes in bodily self-consciousness occurred.

**Conclusions/Significance:**

These data reveal that systematic alterations in the mapping of tactile stimuli occur in a full body illusion and thus establish CCE magnitude as an online performance proxy for subjective changes in global bodily self-consciousness.

## Introduction

The most basic foundations of the self arguably lie in those complex brain systems that represent the body [1]–[5]. This has been explored in research investigating multisensory and sensorimotor bodily mechanisms and their relevance for conscious aspects of processing related to body and self (or bodily self-consciousness: [5]–[9]). An important line of research has studied bodily self-consciousness by investigating the sense of ownership for one's hand [3], [4], [8], [10]–[14]. These experiments manipulated the sense of hand ownership by altering the congruence between multimodal sources of hand-related signals. For example, in the ‘rubber hand illusion’ (RHI), a subject looks at a fake hand that is being stroked by a paintbrush in synchrony with stroking applied to his own (occluded) corresponding hand, positioned a small distance away from the fake hand. Synchronous stroking of the seen fake hand and one's own unseen (real) hand can induce the illusion that the fake hand ‘feels like it's my hand’ (illusory ownership or self-attribution [10], [11], [13]). In the RHI there is also a mislocalization (or drift) of the subject's hand towards the fake hand. Importantly, illusory ownership and drift are much reduced when the stroking is asynchronous [10], [11], [13], [15].

Investigations of the RHI and related studies of the conscious experience of hands and other body parts are very important, but in addition, some authors argue that to achieve a full understanding of bodily self-consciousness we must also investigate its global character [5], [16]–[18]. A fundamental aspect of bodily self-consciousness is that the bodily self is experienced as a single and coherent representation of the entire, spatially situated body, not as a collection of several different body parts [5], [16], [19]. This is also apparent in neurological observations. Although illusory ownership in the RHI and somatoparaphrenia (when neurological patients claim either that their arm belongs to another person or that another person's arm belongs to them [20], [21]) exemplify deviant forms of bodily self-consciousness, they affect body part ownership, or the attribution and localization of a hand with respect to the bodily self, i.e. they are characterised by part-to-whole relationships. This can be contrasted with neurological patients who have illusory perceptions of their full bodies such as in out-of-body experiences and heautoscopy. These states are characterized by abnormal experience with respect to the global bodily self, e.g. a mislocalization and a misidentification of the entire body [22]–[24].

Recent studies [17], [18], [25]–[27] have further demonstrated that global aspects of self-consciousness (self-location and self-identification for the full body) - which are disturbed in neurological patients with autoscopic phenomena - can also be manipulated in healthy individuals by generating multisensory conflicts. In one study [18] subjects viewed their own body from behind via a head-mounted display while their backs were stroked. When the ‘felt stroking’ on the back of the body was congruent with the ‘seen stroking’ on the ‘virtual’ body, subjects showed higher degrees of ownership (or self-identification) for the virtual body, and mislocalized their self to a position outside their bodily borders. The studies on global bodily self-consciousness quantified ownership by verbal or physiological responses [18], [25], [27], or behavioural proxies such as perceived spatial ‘drift’ [18], based on drift measures in the RHI [10]. However these measures do not reveal whether modifications in global bodily self-consciousness are associated with changes in tactile spatial representations. Investigating this aspect is important, as it will reveal whether basic sensory processing of bodily signals is involved in the representation of the bodily self. What is more, the supposed primacy of the tactile sense in self-consciousness [28], [29] generates the prediction that whenever self-location is displaced, an associated change in the mapping of tactile sensations should also occur.

Here we linked the study of global bodily self-consciousness with the measurement of the spatial representation of visuo-tactile stimuli by using the crossmodal congruency task [30]. We hypothesized that this task could function - during the ‘full body illusion’ described above - as an effective measure for probing global aspects of bodily self-consciousness (global ownership and self-location) because the crossmodal congruency effect (CCE) can function as a behavioural index of whether visual and tactile stimuli are functionally perceived to be at the same spatial location. In previous studies of the CCE [12], [30]–[33] the visual and tactile stimuli were presented on the hands (a very recent study tested CCEs with stimuli on feet [34]). Subjects performed worse when a distracting visual stimulus occurred at an incongruent elevation with respect to the tactile (target) stimulus. Importantly, the CCE (difference between performance in incongruent and congruent conditions) was larger when the visual and tactile stimuli occurred closer to each other in space [30]. The CCE has previously been used as a measure of the tactile mislocalisation of touch towards a rubber hand when a fake hand was either aligned or misaligned with subjects' own hands ([12], see also [15]). This measure has a number of advantages: its magnitude is relatively large and it is less susceptible to experimenter expectancy effects than previous behavioural proxies of bodily self-consciousness. Moreover, the congruency task enables the collection of repeated, ‘online’ measurements during manipulations of self-consciousness: this has not previously been done in studies of partial or global bodily self-consciousness.

In the present study we tested whether CCEs – studied so far only for hands – would also be found when viewing one's own body from an external perspective, from two metres behind. Firstly, we studied whether CCEs were modulated by the visual presence or absence of the subject's own body. Secondly, to investigate whether these ‘full body CCEs’ could be associated in a predictable way with changes in bodily self-consciousness, we kept the visual stimulus constant and manipulated self-identification with the virtual body and self-location by employing either synchronous or asynchronous stroking of the back.

## Methods

### Subjects

A total of 35 healthy right-handed subjects took part: 13 (8 males, mean age 24 years) in study 1, 13 (9 males, mean age 26 years) in study 2, and 9 (6 males, mean age 23 years) in the object control study (study 3). Two subjects were excluded from the analyses of study 1 because of chance-level performance in some conditions. Different subjects took part in studies 1, 2 and 3. All subjects had no previous experience with the task or experimental paradigms. All subjects had normal or corrected to normal vision and had no history of neurological or psychiatric conditions.

### Ethics Statement

All subjects gave written informed consent and were compensated for their participation. The study protocol was approved by the local ethics research committee – La Commission d'éthique de la recherche Clinique de la Faculté de Biologie et de Médecine - at the University of Lausanne, Switzerland and was performed in accordance with the ethical standards laid down in the Declaration of Helsinki.

### Materials

**Figure 1:**
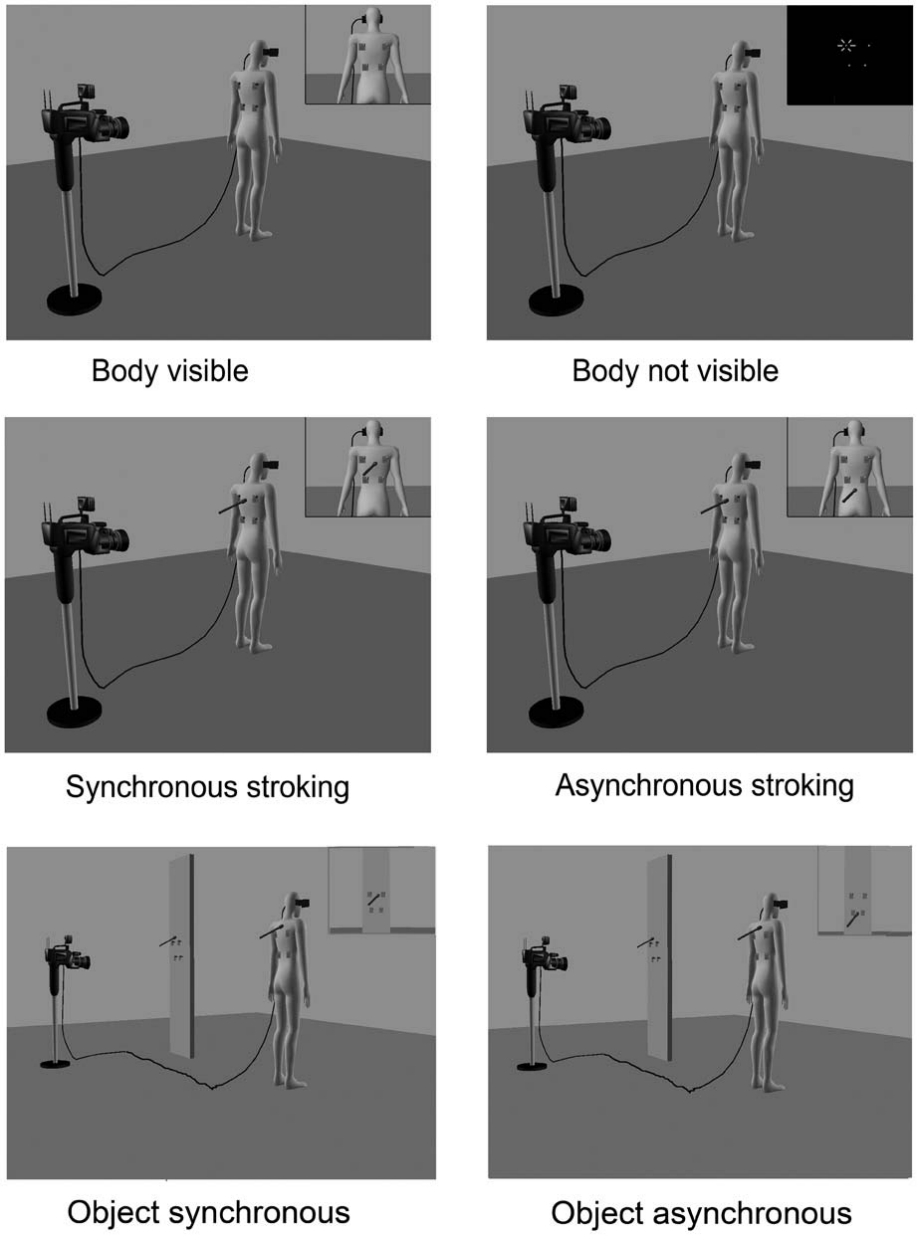
Experimental set-up for different conditions. Subject stood two metres in front of a camera with a 3D-encoder. Four light-vibration devices were fixed to the subject's back, the upper two at the inner edges of the shoulder blades and the lower two 9 cm below. In the object control conditions the lights were attached to a white rectangular metal panel. The small inset windows represent what the subject viewed via the head mounted device. 1. (Upper row) left panel: ‘body visible’ condition; right panel: ‘body not visible’ condition. 2. (Middle row) left panel: synchronous stroking condition; right panel: asynchronous stroking. 3. (Bottom row) - Object control – left panel: synchronous stroking; right panel: asynchronous stroking.

We constructed four ‘light-vibration’ devices, each consisting of a small vibrating motor (Precision MicroDrives shaftless vibration motors, model 312–101, 3V, 60mA, 9000 rpm (150 Hz), 5 g) paired with a single bright light emitting diode (LED; luminance 45 cd/m^2^). The motors had a surface area (the area touching the skin) of 113 mm^2^ and reached maximal rotation speed in approximately 50 ms. The devices were attached to the skin using tape. The two ‘upper’ devices were positioned at the inner edges of the shoulder blades and the two ‘lower’ devices 9 cm below (Fig. 1). Subjects stood with their backs facing a 3D video camera placed 2 metres behind. The video was projected in real time (except for asynchronous blocks, see below) onto a head mounted display (HMD) enabling subjects to view the video in stereoscopic 3D. White noise was presented over headphones to mask any noise from the vibrators, and subjects wore a cloth hood over their heads to occlude vision of their surroundings. The experiment took place under artificial illumination except for the ‘body not visible’ block when the room lights were switched off and the subjects stood in darkness (but could still see the LEDs). During ‘stroking blocks’ the backs (the area spanning the shoulders to waist) of subjects were irregularly stroked, about twice per second by the experimenter with a long wooden stick, and subjects could view the stroking via the HMD. The stroking began one minute before the first vibrotactile stimulus and continued throughout the entire block. In asynchronous blocks a camera delay of 400 msec was introduced (using a delaying device) so that ‘seen stroking’ and ‘felt stroking’ did not correspond.

Stimulus timings were controlled by a program written with E-Prime software. Each trial consisted of a light (LED) flash followed by a vibro-tactile stimulus. The active LED and active vibrating motor were varied randomly and independently from trial to trial. Each trial began with a light flash of 33 msec duration. In study 1, vibro-tactile stimuli were presented 33 msec after the light onset, and for a duration of 100 msec. Note that the vibrator only reached full speed after 50 msec, thus the vibration onset was not exactly at the SoA given. N.B. In studies 2 and 3 the parameters were identical except that vibro-tactile stimuli were presented 233 msec after the light onset. After subjects had responded with a button press there was a 1 second pause before the succeeding trial commenced.

### Procedure

The procedure was identical for all blocks except for those details added below. Subjects were instructed to keep their eyes open and fixate a location in the middle of their backs, as viewed via the HMD. For the first minute of each block no vibrotactile or LED stimuli were presented and subjects were instructed to stand still and wait for the first stimulus. Subjects then had to signal with their right hand, pressing one of two buttons as fast as possible, whether they felt a vibration at the top (an upper device) or at the bottom (a lower device) of their backs (regardless of side), while trying to ignore the light flashes. These responses enabled us to measure reaction times (RTs) and accuracies. At the end of the block (of duration ∼9 mins) global self-localization was measured by first passively displacing the subjects (the experimenter gently guided the subjects - who had their eyes closed - while they took very small steps backwards). They were then asked to walk back to their initial position (while keeping their eyes closed) with normal-sized steps (as in [18]). The distance (the ‘drift’) between the position held during the experimental block and the position indicated by the subject was measured. Self-identification with the seen body and other phenomenological aspects were assessed at the end of each block by a questionnaire adapted from [18]; see Table 1. Subjects took a short break before the subsequent block. All subjects completed a training session (with the body visible and no stroking) prior to the experimental blocks. In study 1 there were 30 trials per condition (same congruent, same side incongruent, different side congruent and different side incongruent) and in studies 2 and 3 there were 25 trials per condition. The order of blocks was counterbalanced across subjects.

**Table 1:** Self-identification Questionnaire.

During the experiment there were times when:	
1	It seemed as if I was feeling the touch of the stick in the location where I saw the virtual body being touched
2	It seemed as though the touch I felt was caused by the stick touching the virtual body.
3	I felt as if the virtual body was my body.
4	It felt as if my (real) body was drifting towards the front (towards the virtual body).
5	It seemed as if I might have more than one body.
6	It seemed as if the touch I was feeling came from somewhere between my own body and the virtual body.
7	It appeared (visually) as if the virtual body was drifting backwards (towards my body).
8	It seemed as though I was in two places at the same time.

Study 1 experimental blocks: (1) Body visible (no stroking) (2) Body not visible (no stroking) – lights in the room were turned off. (3) Synchronous stroking blocks (4) Asynchronous stroking blocks. See figure 1, top and middle panels.

Study 2: All stimulus and procedural details were as described for study 1 except for an increased SOA (233 msec) between the LED and vibro-tactile stimuli. Experimental blocks: (1) Synchronous stroking blocks (2) Asynchronous stroking blocks (3) No stroking blocks (same as ‘body visible’ block in study 1). See figure 1, middle two panels.

Study 3 (object control): All stimulus and procedural details were as described for the synchronous and asynchronous blocks in study 2, except that in ‘synchronous object’ blocks, subjects' backs were stroked with the stick in synchrony with stroking viewed – via the HMD – on a white upright rectangular human-sized metal panel (the object; Fig. 1; bottom two panels). In the ‘asynchronous object’ blocks the subjects' backs were again stroked with the stick but a delay was added to the visual display presented on the HMD (as described in study 2) so that the ‘felt stroking’ was asynchronous with respect to the seen stroking on the object. In the object blocks the vibrators were attached to the backs of subjects, as described previously, but the LEDs were attached to the object and were placed at the same height from the ground and at the same relative distances as the vibrators on the subjects' backs.

### Statistical analysis

Trials with incorrect responses and trials in which subjects failed to respond within 1500 msec were discarded from the reaction time (RT) analysis (following the method of [30]). As a result an average of 4.8% of trials per subject were discarded. The mean RTs and the drift (self-location) measures (calculated relative to initial position  = 0) were normally distributed (Kolmogorov-Smirnov test for normality) and were analyzed using two-tailed repeated measures analyses of variance (ANOVA) and two-tailed t-tests, respectively. The questionnaire scores were analyzed using a non-parametric test (Wilcoxon matched pairs test). The significance (alpha) level used was 0.05.

For study 1, RT and accuracy data were analysed using a repeated measures ANOVA with three factors: body (body visible/not visible), side (same/different) and congruency (congruent/incongruent). Mean RT and errors for all conditions are shown in table 2. To examine the effect of stroking type, a separate repeated measures ANOVA was run with factors stroking type (asynchronous/synchronous), side (same/different) and congruency (congruent/incongruent). For study 2 and study 3, RT and accuracy data were again analysed using a repeated measures ANOVA with the factors stroking type, side and congruency. We mainly focus on the RT data rather than accuracy, as this has been shown to be more sensitive [12], [15], [33].

**Table 2:** Mean reaction time and percentage of errors for tactile targets in Studies 1–3 as a function of the visual distractor with respect to the target, the distractor's congruence with the target and the experimental condition.

Target-distractor congruence	Position of distractor	Reaction Time (ms)	Error (%)
*Study 1- Body Visible*			
Congruent	same	528 (24)	4 (1)
	different	575 (28)	7 (2)
Incongruent	same	668 (40)	12 (4)
	different	630 (27)	10 (3)
*Study 1- Body Not visible*			
Congruent	same	558 (33)	4 (2)
	different	550 (38)	4 (1)
Incongruent	same	577 (40)	8 (2)
	different	570 (37)	6 (1)
*Study 1- Body Synchronous*			
Congruent	same	655 (44)	13 (3)
	different	690 (49)	18 (3)
Incongruent	same	773 (45)	36 (4)
	different	760 (50)	27 (4)
*Study 1- Body Asynchronous*			
Congruent	same	698 (48)	17 (4)
	different	746 (45)	13 (2)
Incongruent	same	822 (34)	37 (8)
	different	782 (35)	25 (3)
*Study 2- Body Synchronous*			
Congruent	same	543 (22)	12 (2)
	different	627 (32)	19 (3)
Incongruent	same	651 (31)	28 (5)
	different	643 (33)	22 (3)
*Study 2- Body Asynchronous*			
Congruent	same	587 (34)	14 (2)
	different	616 (30)	22 (2)
Incongruent	same	636 (29)	31 (4)
	different	634 (33)	24 (3)
*Study 3 - Object Synchronous*			
Congruent	same	646 (65)	13 (2)
	different	669 (62)	16 (3)
Incongruent	same	695 (58)	36 (12)
	different	710 (55)	36 (9)
*Study 3 - Object Asynchronous*			
Congruent	same	698 (56)	11 (2)
	different	655 (53)	10 (3)
Incongruent	same	690 (46)	33 (12)
	different	701 (61)	33 (10)
*Study 3 - Body Synchronous*			
Congruent	same	615 (50)	8 (3)
	different	674 (44)	11 (2)
Incongruent	same	795 (49)	39 (10)
	different	726 (31)	41 (9)
*Study 3 - Body Asynchronous*			
Congruent	same	698 (33)	6 (2)
	different	749 (44)	14 (3)
Incongruent	same	765 (28)	37 (9)
	different	800 (51)	31 (6)

Standard errors are in parentheses.

## Results

### Results of Study 1

**Figure 2:**
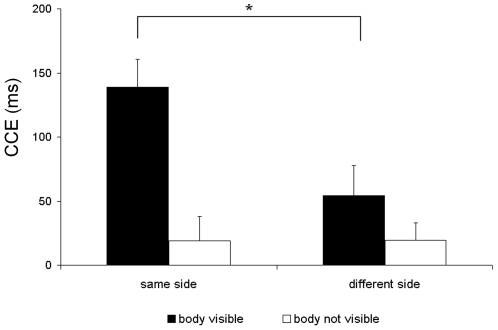
CCE in study 1 – ‘body visible’ and ‘body not visible’ conditions. Mean congruency effects in reaction time (RT) in milliseconds (RT in incongruent trials minus RT in congruent trials) in Study 1 for ‘body visible’ and ‘body not visible’ conditions. Error bars show standard errors of the mean.

Figure 2 plots the size of the full body CCE (reaction time in incongruent trials minus RT in congruent trials) for the body visible and body not visible conditions. In the body visible condition the CCE was larger when the light appeared on the same side as the tactile stimulus, compared to when it appeared on the different side. The ‘body not visible condition’ did not show these effects on the size of the CCE. Statistical analysis revealed a significant main effect of congruency (F_1,10_ = 15.25; p = 0.003), a significant interaction between body and congruency (F_1,10_ = 21.63; p = 0.001), a significant interaction between side and congruency (F_1,10_  = 7.66; p = 0.02) and crucially, a significant three-way interaction between body, side and congruency (F_1,10_  = 10.13; p = 0.01). Planned comparisons between same and different side CCEs for body visible and body not visible conditions revealed that the CCE was significantly larger for the same side compared to the different side visual presentation when the body was visible (t_1,10_ = 3.22; p = 0.009) but not when the body was not visible (t_1,10_ = 0.83; p>0.05). The error rates showed a similar pattern of modulation by congruency and side. There was a significant main effect of congruency (F_1,10_ = 8.36; p = 0.016) but no other main effects or interactions. The congruency effect in error rate when the body was visible was 8% for the same side and 3% for the different side. When the body was not visible the congruency effect was 4% for same side and 2% for different side.

**Figure 3:**
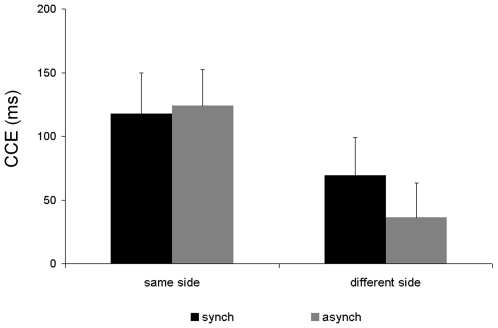
CCE in study 1 – synchronous and asynchronous stroking conditions. Mean congruency effects in reaction time in milliseconds (RT in incongruent trials minus RT in congruent trials) in Study 1 for synchronous and asynchronous conditions.

The difference in the size of the CCE for same and different side light presentation was similar for synchronous and asynchronous conditions (figure 3). Statistical analysis revealed a significant main effect of congruency (F_1,10_  = 18.06; p = 0.002) and a significant interaction between side and congruency (F_1,10_  = 8.82; p = 0.014). No other effects reached significance (p>0.05), and there was no significant three-way interaction between stroking, side and congruency. The error rates again showed a similar pattern of modulation by congruency and side. There was a significant main effect of side (F_1,10_  = 30.83; p = 0.000); a significant main effect of congruency (F_1,10_  = 39.3; p = 0.000) and a significant interaction between side and congruency (F_1,10_  = 14.18; p = 0.004). No other terms reached significance. The congruency effect in error rate for synchronous stroking was 23% for the same side and 9% for the different side. For asynchronous stroking the congruency effect was 20% for same side and 12% for different side. These error rates are somewhat higher than those found in previous CCE studies and this is probably due to two factors: applying the vibrations to the skin on the back (which is less sensitive than the skin on the fingers) and applying the stroking at the same time as the vibrations (which may have made the task more difficult by introducing tactile ‘noise’).

No significant effects (p>0.05) of stroking type were found for self-location – the size of the drift in self-location did not differ for synchronous and asynchronous conditions. Further, there were no significant differences in the questionnaire ratings (see table 1 for list of questions) between these conditions.

As predicted, we found that the CCE is present for the full body, and is larger when subjects see their body (as compared to when they do not see their body). Our second prediction was not confirmed: stroking (synchronous versus asynchronous) neither modulated the CCE, nor did it modify self-location or self-identification. The lack of a stroking effect on self-location and self-identification (question 3) is, at first sight, at odds with the findings of a previous study [18] that found a significant increase in both measures during synchronous stroking. This may have been because in the present set-up we added a second visuo-tactile mismatch (LED versus vibration) to the visuo-tactile mismatch already present due to stroking, thus the stimuli in the congruency task may have interfered with mechanisms related to self-location and self-identification. In particular, our stroking conditions may have been ‘more synchronous’ (or ‘less asynchronous’) as a result of the introduction of the short interval LED-vibro-tactile stimuli. A complex range of temporal, spatial (and cognitive) factors determines the weighting of each unisensory input during multisensory integration [35]–[37]. It is therefore possible that the particular combination of visual and tactile signals that were present in the experimental conditions in study 1 may have affected the visuo-tactile integration involved in the computation of self-location. The stimulus onset asynchrony (SOA) of 33 msec in study 1 was chosen based on previous studies on CCEs [30], [33] which reported maximal CCEs for an SOA of ∼30–100 msec. It may be that processes related to visuo-tactile integration during the combined presentation of the LED/vibro-tactile stimuli and the seen/felt stroking were different to the visuo-tactile integration that occurs when the LED/vibro-tactile stimuli are presented alone.

We therefore ran a second study with a different (increased) SOA between the LED and vibro-tactile stimuli. In order to maximise temporal asynchrony between these stimuli, but potentially retain a CCE, we chose an SOA of 233 msec based on results showing that the CCE is still present for SOAs of ∼200 msec [33]. Stimuli were presented in three different experimental blocks – synchronous, asynchronous and no stroking blocks - as in study 1.

### Results of Study 2

**Figure 4:**
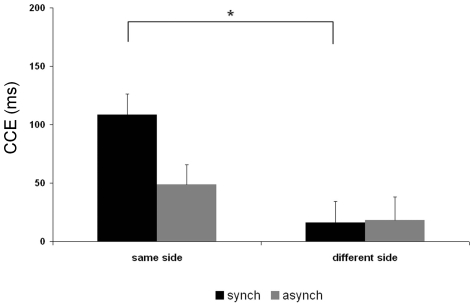
CCE in study 2 – synchronous and asynchronous stroking conditions. Mean congruency effects in reaction time in milliseconds (RT in incongruent trials minus RT in congruent trials) in Study 2 for synchronous and asynchronous conditions. Error bars show standard errors of the mean.

With an SOA of 233 msec, we found that the type of stroking modulates the CCE. In the synchronous condition the CCE was larger when the light appeared on the same side as the tactile stimulus compared to when it appeared on the different side, whereas the CCE during asynchronous stroking did not differ for same and different side light presentations (see figure 4). The ANOVA revealed a significant main effect of side (F_1,12_  = 9.10; p = 0.011), congruency (F_1,12_  = 15.80; p = 0.002), a significant interaction between side and congruency (F_1,12_  = 13.40; p = 0.003), and crucially, a significant three-way interaction between stroking type, side and congruency (F_1,12_  = 11.30; p = 0.006). Planned comparisons between same and different side CCEs for synchronous and asynchronous conditions revealed that the CCE was significantly larger for the same side than different side in the synchronous condition (t_1,12_ = 4.01; p = 0.002), but not in the asynchronous condition (t_1,12_ = 2.17; p>0.05). The error rates showed a similar pattern of modulation by congruency and side. There was a significant main effect of congruency (F_1,12_  = 21.69; p = 0.001) and a significant interaction between side and congruency (F_1,12_  = 60.46; p = 0.000). There were no other significant main effects or interactions. The congruency effect in error rate for synchronous stroking was 16% for same side and 3% for different side. For asynchronous stroking the congruency effect was 18% for same side and 3% for different side.

**Figure 5:**
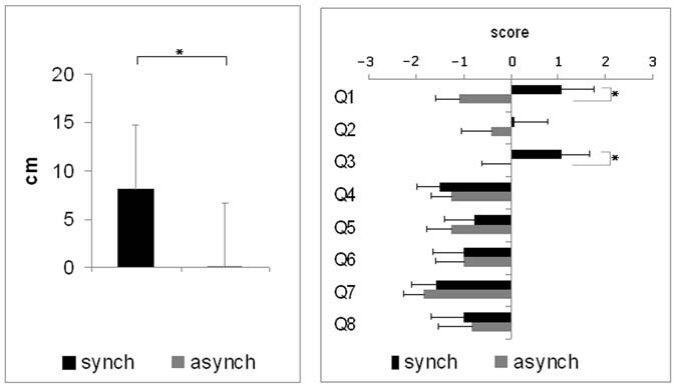
Drift and questionnaire scores in study 2. 1. (Left inset) Drift measured in cm for synchronous and asynchronous conditions on the posterior-anterior axis (Study 2). 2. (Right inset) Score on the “self-identification questionnaire” (Study 2) as adapted from [10].

In the synchronous condition the subjects showed a mean drift in self-location of 8.1 cm towards the virtual body, whereas in the asynchronous condition the mean drift was 0.1 cm (Figure 5). The size of the drift in the synchronous condition was significantly different from the drift in the asynchronous condition (t_1,12_ = 2.21; p = 0.047; two-tailed t-test). For the questionnaire data, statistical analysis revealed significant differences between the synchronous and asynchronous conditions only for questions 1 and 3. Participants gave a significantly higher positive rating in the synchronous condition compared to the asynchronous condition for question 1 (“It seemed as if I was feeling the touch of the stick in the location where I saw the virtual body being touched”) evaluating touch (z = 2.8; p = 0.005) and for question 3 (“I felt as if the virtual body was my body“) evaluating self-identification (z = 2.3; p = 0.020), see Table 1 and Figure 5.

Study 2 revealed that the CCE differs between synchronous and asynchronous stroking, and study 1 showed that CCEs are found when the LEDs are presented on a body but not when the body is not visible. Study 3 was carried out to further understand these effects. In order to determine whether the modulating effect of stroking is specific to the case where a human body is viewed or could also be found when an inanimate object is stroked, we ran a final control experiment with nine subjects who viewed (via the HMD) synchronous and asynchronous stroking on an object or on their body while stroking was applied to their backs, as before.

### Results of Study 3

**Figure 6:**
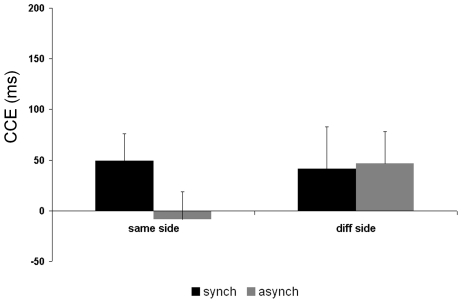
CCE in study 3 – Object control. Mean congruency effects in reaction time in milliseconds (RT in incongruent trials minus RT in congruent trials) in the object control study (Study 3) for synchronous and asynchronous conditions.

Figure 6 plots the size of the CCE for same and different sides for synchronous and asynchronous stroking for the object control condition. For the object condition there was no difference in the size of the CCE for same side versus different side visual presentation during either synchronous stroking or asynchronous stroking. The ANOVA did not reveal any significant main effects: stroking type (F_1, 8_ = 0.04, p = 0.846); side (F_1, 8_ = 0.01, p = 0.921); congruency (F_1, 8_ = 4.43, p = 0.068), or any significant interactions: stroking type×side (F_1, 8_ = 1.57, p = 0.246); stroking type×congruency (F_1, 8_ = 0.60, p = 0.460); side×congruency (F_1, 8_ = 0.452, p = 0.520); stroking type×side×congruency (F_1, 8_ = 1.17, p = 0.311). We replicated the previous result for the ‘body’ condition in this new sample of subjects, i.e. the effects confirmed those of study 2: during synchronous stroking the CCE was significantly larger when the light appeared on the same side as the tactile stimulus compared to when it appeared on the different side (t_1,8_ = 2.60; p = 0.031), whereas the CCE during asynchronous stroking did not differ for same and different side light presentation (t_1,8_ = 0.39; p>0.05).

Analysis of the error rates for the object control showed that error rates were lower for the asynchronous condition than the synchronous condition: there was a significant main effect of stroking type (F_1, 8_ = 5.61, p = 0.045). There were no other significant main effects: side (F_1, 8_ = 0.179, p = 0.683); congruency (F_1, 8_ = 5.14, p = 0.053), and no significant interactions: stroking type×side (F_1, 8_ = 0.278, p = 0.612); stroking type×congruency (F_1, 8_ = 0.037, p = 0.852); side×congruency (F_1, 8_ = 0.026, p = 0.876); stroking type×side×congruency (F_1, 8_ = 0.163, p = 0.697).

There was no significant difference in the mean drift between the synchronous object and asynchronous object conditions (t_1,8_ = 0.41; p>0.05). For the questionnaire data, there was a significant difference between ratings of the self-identification question (“I felt as if the object was my body“) for the synchronous and asynchronous object blocks (z = 2.20; p = 0.03), however the ratings were barely, or not even positive (4.1 for synchronous and 2.9 for synchronous on a scale where rating 4 is neither positive nor negative, i.e. ‘zero’).

## Discussion

Linking the study of the spatial representation of visuo-tactile cues with manipulations of bodily self-consciousness, we report three principal findings. First, we show that crossmodal congruency effects are stronger when visual distractors are presented on one's seen body compared to when they are presented in the dark. Second, full body CCEs are larger during synchronous stroking than during asynchronous stroking of one's back, and depend on the temporal delay between the vibro-tactile and LED stimuli. Note that this effect of stroking on the CCE is not found when the LEDs and the stroking are viewed on a human-sized object instead of the body. Third, both full body CCEs and measures of bodily self-consciousness are modulated by visuo-tactile stimulation (type of stroking): In the synchronous stroking condition CCEs are larger, the drift towards the seen body is greater and the questionnaire ratings of self- identification with the seen body are higher compared to in the asynchronous condition. These novel data reveal ‘body-related’ and ‘self-related’ CCEs and suggest that under the conditions used in the present study the full body CCE is associated with key components of bodily self-consciousness, i.e. ‘what I experience as my body’ and ‘where I experience my body to be.’

By demonstrating CCEs for the full body our data extend the findings of previous studies that have used this paradigm for body parts, usually hands [12], [15], [30]–[32]. In the present study, the CCEs were larger when the visual distractors appeared on the same side (e.g. right side) of the body as the vibro-tactile targets than when the distractors appeared on the other body side (e.g. left side). This demonstrates that even for the full body, the magnitude of the CCE is modulated by the perceived spatial distance between the tactile target and the visual distractor (as previously observed for hand CCEs [12], [15], [30], [32]). Such effects were absent when the stimulus display did not show the subject's body, and when an object was viewed in the place of the body, even though the visual stimuli (LEDs) were still visible and in the same spatial configuration. It is notable that the full body CCEs were observed even though subjects had an external, implausible, view of their body (they viewed the back of their body, which cannot be directly seen), standing two metres in front.

Our findings are also compatible with data reporting visual capture of touch when lights were presented on fake hands [12], [15]. CCEs have also been measured for shadows of hands [32] and even for photographs of hands presented via video monitors [31]. More generally, this is consistent with studies [7], [38]–[43] demonstrating how the sight of one's own body parts can influence tactile perception, in some cases even with views of body parts (e.g. the neck) that cannot be directly seen [42], [43].

A second major finding of the present study is that, as predicted, the CCE was larger during synchronous than asynchronous stroking (study 2). Modifying visual-somatosensory congruence by employing different types of stroking enabled us to manipulate whether or not subjects felt as if they were looking at their own body, as indicated by the questionnaire data (see below). The CCE was only modulated by stroking when we introduced a larger temporal asynchrony between the LED and vibro-tactile stimuli. It is well known that multisensory integration is strongly influenced by the temporal relations between stimuli [33], [35]–[37]. Our experiment incorporated two ongoing visuo-tactile ‘events’: the seen and felt stroking, and the combined LED-vibro-tactile stimuli. We argue that when the LED-vibro-tactile stimuli were made more asynchronous (by introducing the larger SOA), this may have influenced the differential weighting of all visual and tactile stimuli present, and therefore have affected how the felt stroking and seen stroking were integrated. Alternatively, it is also possible that in study 1 when the SOA was smaller, i.e. when the LED and vibration signals were more synchronous, this interfered with the stroking by rendering the asynchronous condition ‘less asynchronous’.

Our third major finding is that both full body CCEs and measures of bodily self-consciousness are modulated by visuo-tactile stimulation (type of stroking). During the synchronous condition there was (1) a larger CCE, (2) a greater degree of self-identification (as shown by Q3 in the questionnaire data) and (3) a larger drift in self-location towards the virtual body (as shown by the drift measure) compared to the asynchronous condition. This suggests that a greater degree of visual capture of tactile location occurs in the experimental condition in which there is a greater degree of self-identification for the seen body.

The present data suggest that the tactile stimuli are mislocalised to a different place in external space (towards the seen body in the synchronous condition) because the localisation of the body in space is biased towards the seen body (as measured by the drift and questionnaire) in the condition in which the CCEs are larger. In the synchronous condition it is not merely that the CCE is larger than in the asynchronous condition: there is also a greater difference between the same side and different side CCEs in the former condition. This side effect is likely to be a due to a change in the spatial perceptual representations because if the touch is represented as being closer in space to where the body (and LEDs) are seen then we would expect the difference between same and different side CCEs to be larger. This is because when the virtual body and the real body are perceived as being closer the distance between a given tactile stimulus and a *different side* visual distractor is greater than that between a tactile stimulus and a *same side* distractor.

It should be noted that visual capture of touch is not the only possible explanation for the increased CCE. Alternative explanations for differences in CCE magnitude have been discussed in depth by Spence and colleagues [44]. One possibility is that seeing the visual stimuli on the body increases their task relevance. While this could explain the results of study 1 where we compare CCEs when the body was or was not seen, it is not clear how effects of task relevance could account for the results obtained in study 2 (where we found that the different types of stroking modulated CCEs differently). Response bias – where, e.g. incongruent ‘up’ stimuli prime the ‘up response’ - is another factor thought to contribute to CCE magnitude [30], [33], [44] - but it cannot explain the differential side effects found in all three studies. An interesting alternative explanation is that the difference in CCEs is not due to tactile recoding but to visual recoding. It could be that seeing the visual stimuli on the body causes these distant visual stimuli to be recoded so that they are made equivalent to near visual stimuli in their effects [44], [45]. This could explain the results of study 1, as the sight of one's body could cause the recoding of the visual stimuli so that they are represented as being closer to the tactile stimuli. One might also argue that in study 2, the synchronous stroking increased this visual recoding effect (compared to asynchronous stroking) or otherwise the asynchronous stroking decreased it. The difference in CCEs we report can only show that tactile and spatial stimuli are perceived as being closer to each other (in the synchronous condition) – this finding cannot by itself show whether it is touch or vision that is remapped. Nevertheless, given that there is a change in self-location - as measured by the spatial drift - towards the seen body (i.e. towards the visual stimulus) in the synchronous condition, we suggest that it is touch rather than vision that is mislocalised.

The self-related aspects of the CCE appear to be not simply an effect of seeing *a* body, but of identifying with the seen body, and having an altered self-location (biased towards the spatial location at which the body is seen). This is further evidence for the predicted [28], [29] strong association between self-consciousness and the tactile sense. These findings are unlikely to be due to a difference in the level of attention between the conditions, since stimulus-based differences were minimized between the synchronous and asynchronous stroking conditions. Further, our finding that there was no CCE (no significant effect of congruency and no interaction between synchrony, side of visual distractor and congruency) in the object control condition suggests that the effect of stroking on the CCE is specific to the case where the stroking is applied to a human body.

The congruency task we employed has several advantages for use in studies of bodily self-consciousness: firstly, it is an online measure of self-location and hence can be measured *during* full body illusions. This task is thus arguably an improvement on methods used both in previous studies of partial (body part) ownership [10], [11], [13] and studies of global ownership/self-identification [18], [25], since these measured behavioural/physiological proxies of bodily self-consciousness *after* the stroking period. Moreover, the magnitude of the CCE is relatively large, and multiple repeated measures can be obtained; this was not the case during most previous manipulations (although note that repeated CCE measures were collected in studies of the ‘fake hand effect’ [12], [15]). Performance in the congruency task is also likely to be much less susceptible to observer biases that may have affected self-location and questionnaire measures in previous studies [18], [25]. The present CCE task is relatively simple, involving only speeded, forced choice, perceptual judgements - no high level introspective reflections (as questionnaires require) - and is thus suitable for use in patient studies and even in animal studies.

Pavani and colleagues [12] used the CCE to investigate tactile spatial perception when vibrations were applied to subjects' hands, and lights (LEDs) were presented on rubber hands. In this study, the CCE was present only when the rubber hands had the same posture as the real hands, and in this case subjects were more likely to report feeling the touch at the location of the rubber hands (see also [15]). Despite the importance of these earlier CCE studies [12], [15], we argue that they have certain limitations in terms of investigating bodily self-consciousness. Firstly, these studies did not directly manipulate self-attribution (e.g. by stroking) *during* the CCE measurements, but only after. Secondly, these (and other [10]–[13], [32], [40]) authors concentrated on the investigation of the representation of body parts, but did not manipulate aspects related to global bodily self-consciousness such as self-location and self-identification of the full body [16]. These global aspects are altered in the full body illusion [17], [18], [25], [27] and in autoscopic phenomena (see, e.g. [22]–[24], [46]. During heautoscopy, patients see a second own (illusory) body in extrapersonal space and self-location is either experienced at the position of the physical body or at the position of the illusory body, or at both. Moreover, self-location may change the experienced position (between the position of the illusory and the physical body) and this may occur in rapid alternation [22], [23]. Patients self-identify either with the illusory body, the physical body, or with both in alternation [22], [47], [48]. Altered self-location and self-identification with an illusory body at an extracorporeal position are strongly present in OBEs: the self is experienced as localized outside one's bodily borders (disembodiment). In OBEs, self-location is never at the position of the physical body. The present data show that the previously described [17], [18], [25], [27] changes in self-location and self-identification are - under certain experimental conditions - associated with changes in the CCE, and hence with changes in the mapping of tactile stimuli.

Since stimuli were applied only to the backs of the subjects in the present study it is possible that non-stimulated body parts were not affected by the stroking manipulation and thus that the measured effects were not global. However, as argued elsewhere [16], we believe that the present experimental manipulations did enable us to investigate global/full-body representations. Firstly, CCEs were associated with changes in self-identification (and thus more global changes than changes in self-attribution measured in the RHI). Secondly, the CCEs were larger in the condition (synchronous condition) in which the change in self-location was greater. Interference with more global aspects of bodily processing is also likely given back (trunk) stroking because tactile receptive field properties differ substantially between neurons encoding the trunk (large and bilateral receptive fields) and those encoding hands or feet (small and unilateral receptive fields) [49], [50]. It would be interesting and important for future studies to investigate - in detailed follow up experiments - whether the effects of stroking applied on the trunk (as done here) generalizes to non-stimulated body parts, e.g. the hands.

In conclusion, the present data reveal full body-related CCEs, and ‘self-related’ CCEs, the latter demonstrating that the magnitude of the CCE is associated with ‘what I experience as my body’ and ‘where I experience my body to be’. The experimental manipulation of self-identification (via stroking) combined with the measurement of self-location estimates (CCEs) enabled us to characterize bodily self-consciousness in terms of underlying multisensory mechanisms, thereby extending recent data [17], [18], [25], [27] on global bodily self-consciousness. The present study reveals that systematic alterations in the mapping of tactile stimuli occur in the full body illusion, and thus establishes CCE magnitude as an online performance proxy for subjective changes in bodily self-consciousness.
